# Polystyrene Nanoplastics in Aquatic Microenvironments Affect Sperm Metabolism and Fertilization of *Mytilus galloprovincialis* (Lamark, 1819)

**DOI:** 10.3390/toxics11110924

**Published:** 2023-11-11

**Authors:** Martina Contino, Greta Ferruggia, Stefania Indelicato, Roberta Pecoraro, Elena Maria Scalisi, Antonio Salvaggio, Maria Violetta Brundo

**Affiliations:** 1 Department of Biological, Geological and Environmental Sciences, University of Catania, Via Androne 81, 95124 Catania, Italy; greta.ferruggia@phd.unict.it (G.F.); stefania.indelicato@phd.unict.it (S.I.); roberta.pecoraro@unict.it (R.P.); elenamaria.scalisi@unict.it (E.M.S.); mariavioletta.brundo@unict.it (M.V.B.); 2 Zooprophylactic Institute of Sicily “A. Mirri”, Via Gino Marinuzzi, 3, 90129 Palermo, Italy; antonio.salvaggio@izssicilia.it

**Keywords:** bivalves, polystyrene, fertilization, gametes, pollution

## Abstract

The continuous and unregulated discharge of wastes and pollutants into the aquatic environment has required constant monitoring of the risks incurred by aquatic ecosystems. Alarmism arises from plastic pollution as larger artifacts release nanoscale fragments that can contact free-living stages such as gametes, embryos, and larvae. Specifically, the interaction between spermatozoa, released in water in externally fertilizing species, and the surrounding microenvironment is essential for successful fertilization. Activation and kinematics of movement, proper maintenance of ionic balance, and chemotactism are processes highly sensitive to even minimal perturbations caused by pollutants such as polystyrene nanoplastics. Spermatozoa of *Mytilus galloprovincialis* (*M. galloprovincialis*), an excellent ecotoxicological model, undergo structural (plasma membrane ruptures, DNA damage) and metabolic (reduced motility, fertilizing capacity) damage upon exposure to 50 nm amino-modified polystyrene nanoplastics (nPS-NH_2_). Nanoplastics of larger diameter (100 nm) did not affect sperm parameters. The findings highlighted the negative impact that plastic pollution, related to nanoparticle diameter and concentration, could have on sperm quality and reproductive potential of organisms, altering the equilibrium of aquatic ecosystems.

## 1. Introduction

Species with external spawning, such as sessile marine invertebrates (bivalves), release their gametes into a marine microenvironment that is now perturbed by various stressors due to climate change (reduced PH, increased temperature) [1,2,3] and growing concentrations of various emerging contaminants (drugs, plastics, additives), concentrated over decades due to human negligence [4,5,6]. Currently, the scenario emerging from the literature is alarming due to the uncontrolled spread of plastics [7,8,9]. Among them, the most prevalent correspond to nanoplastics, i.e., spheres <1 mm in size, originating from physical (photocatalysis, action of waves, current, attrition with sediments), chemical (enzymatic action), or biotic (action of microorganisms) degradation processes of plastic waste (cosmetics, packaging, containers, paints) [10,11,12,13]. The hazard of nanoplastics is correlated to several characteristics, including their ability to adsorb contaminants from the surrounding aquatic environment and to transport carcinogenic molecules used as additives, including plasticizers such as bisphenol A, pharmaceuticals, and metals, mainly due to the presence of chemical functional groups (amino or carboxy groups) [14,15,16,17]. This mechanism defined as the “Trojan-Horse effect” greatly increases the hazard of nanoplastics [18,19,20]. Although the behavior of nanoplastics in some situations can be influenced by various factors such as salinity, pH, and organic matter content (leading to the formation of larger aggregates). In general, the large surface area and high area/volume ratio increase interactions with biological membranes, becoming a risk for organisms [21,22].

Polystyrene (PS), phenylethane for I.U.P.A.C., is one of the most important chemical elements in the production of plastics due to its properties such as malleability, thermo plasticity, and an amorphous nature, making it optimal to produce many products used during daily life such as food containers and jars, disposable tableware, packaging for the production of paints, and scrubs for cosmetics [23,24,25]. PS has been found in all major environmental matrices although its exact quantization is not always easy to obtain [26]. With polystyrene particles traveling from the coast over long distances to other parts of the world (Mediterranean and Adriatic Seas, Korea, China), even to the most remote areas such as polar or desert regions, an overabundant presence of polystyrene particles of various sizes has been recorded [27,28]. Kwon et al., 2015, analyzed more than 500 sand and seawater samples taken from more than 21 nations. The highest concentrations of PS were observed in waters near the most industrialized and populated areas of the planet such as the United States where these ranged from 6.9 to 30.4 μg/L [29]. The same authors, in 2017, also found styrene in the deep waters of the Pacific Ocean with a concentration between 0.31 and 4.31 μg/L. However, it is estimated that these concentrations are destined to increase [30].

Many studies highlighted the effects of chronic exposure to polystyrene nanoplastics on the metabolism and survival of aquatic organisms and, in particular, species considered sentinel such as *M. galloprovincialis*, identifying, through a multi-biomarker approach, increased genotoxicity and oxidative stress in different organs such as the digestive gland, gills, and immune system [31,32,33]. Furthermore, the synergistic toxic effect due to the presence of nanoplastics in an aquatic environment already affected by rising temperatures and decreasing dissolved oxygen could contribute to climate change [34].

The assessment of seawater quality plays a key role for the prediction of the effects of pollutants not only on gametes, fertilization, and the early stages of embryonic development, but also on the equilibrium and stability of ecosystems. It is well known, in fact, that sudden variations in the number of individuals in a population, in addition to impacting the population’s ability to survive, also lead to alterations in interspecies relationships, e.g., prey–predator, and in animal spatial distribution [35]. For this reason, bioassays were designed to explore the impact of contamination by potentially toxic substances, investigating any biochemical, metabolic, molecular, and structural alterations implicated in fertilization failure [36]. In this context, *Mytilus galloprovincialis* (Lamark, 1819), and in general the bivalves, assume an interesting role as a model organism, due to their almost ubiquitous distribution, easy retrieval, and easy handling of gametes and embryos in the laboratory [37,38]. While several studies in the literature focused on the possibility of bioaccumulation of nanoplastics in different organs, including the gonads, few experiments have investigated the effect of polystyrene micro- and nanoplastics on the physiology and metabolism of gametes once released into water. In general, as shown in Table 1, the plastics result in a worsening of the fundamental parameters of the semen, causing a reduction in the fertilization rate. In addition, amino-modified nanoplastics appear to be more toxic than carboxy-modified ones.

The present study was conducted to understand whether contaminants may be responsible for the change in the optimal parameters that allow, after the release of gametes, the activation of spermatozoa with the consequent attainment of the egg cell; the maintenance of plasma membrane integrity; and the normal fertilization and the physiological embryonic development that begins with the activation of the egg cell (lifting of the fertilization membrane and extrusion of the second polar globule). For this reason, the present study investigated the effects of amine-modified polystyrene particles (100 nm and 50 nm nPS-NH_2_) on sperm parameters of *M. galloprovincialis.* Following an acute exposure (30 min) to increasing concentrations of polystyrene, conforming to those measured in the environment, different parameters were evaluated: motility, viability, DNA fragmentation, and oxidative stress. In addition, the subsequent fecundating capacity of these gametes was monitored, with a focus on the earliest stages of embryonic development. The aim was to identify, in parallel to the potential anomalies induced, the potential correlation between the diameter and the toxicological profile of the particle tested.

## 2. Materials and Methods

### 2.1. Preparation of Solutions

The selected amino-modified polystyrene was purchased in the form of nanospheres with diameters of 100 and 50 nm (Sigma-Aldrich, St. Louis, MO, USA). The nPS-NH_2_ were fluorescent (100 nm: 481–644 nm excitation/emission; 50 nm: 358–410 excitation/emission), thus easily identifiable through a fluorescence microscope (Nikon ECLIPSE Ci), using the FITC filter to detect green fluorescence (100 nm) and the DAPI filter for blue fluorescence (50 nm). They also had a density between 1.03 and 1.07 g/cm^3^ like that of nPS-NH_2_ found in the sea [44]. Concentrations suggested by other studies and used in other animal models such as *Artemia franciscana* and *Brachionus plicatilis* were tested [45,46]. The concentrations chosen were 1 µg/L, 10 µg/L, 20 µg/L, 50 µg/L, and 100 µg/L. Solutions were prepared in fresh seawater (FSW) previously filtered with 0.20 µm filters. All solutions were sonicated for 2 min (Sonoplus) to avoid the aggregation of nanoparticles.

### 2.2. M. galloprovincialis Gametes Collection and Exposure

**Figure 1:**
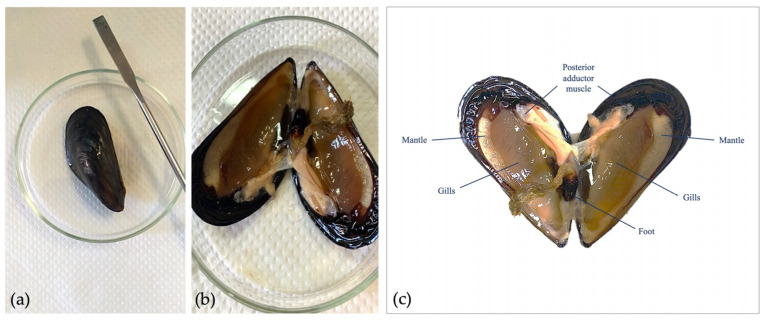
Gamete collection of *M. galloprovincialis*. (**a**) Adult organism; (**b**) opening of valves for biopsy collection; (**c**) anatomy of adult organism.

Mussels, 4–5 cm long, were sampled in the Mediterranean Sea and were transferred to the laboratory of Biotechnology of Reproduction (University of Catania, Italy) and acclimatized in static tanks containing aerated artificial sea water [47], with pH 7.9–8.1 and 36 ppt salinity (1 L/animal), at 16 ± 1 °C. They were opened by cutting the adductor muscle, and sperm samples were obtained via biopsy collection from gonadal tissue through a Pasteur pipette to obtain a concentrated sample, to which 1 mL of FSW was added to activate the spermatozoa (Figure 1). Optimal samples were selected following activation in seawater after about 30 s and subsequent evaluation of motility using microscopy. Only samples with a motility above 80% were selected for the experiment. An aliquot was diluted in distilled water (1:1000, *v*/*v*) to estimate sperm concentration using Burker’s counting chamber. Then, the sample was diluted in 1 mL of working solution, previously prepared, to obtain the concentration of 5 mil spz/mL. After an acute exposure of 30 min at room temperature, sperm parameters were evaluated. Three replicates were performed for each test.

### 2.3. Sperm Motility

To assess sperm movement, the CASA plugin, installed on ImageJ1.44, was used, following the instructions of Wilson-Leedy and Ingermann (2007) [48], with which various parameters were monitored: percentage of mobile spermatozoa, Velocity Curvilinear (VCL), Velocity Average Path (VAP), Velocity Straight Line (VSL), Linearity (LIN), Wobble (WOB), and Beat Cross Frequency (BCF). Videos of 10 s each were uploaded to ImageJ, acquired at a resolution of 1920 × 1080 using a video camera (Nikon Y-TV55), connected to an optical microscope (Nikon Eclipse E-200). Slides were set up by placing 5 µL of the sample, covered with a 24 × 24 coverslip, and observed, after 30 s to allow stabilization of the liquid inside the chamber, at 40× magnification. 

### 2.4. Integrity of Plasmatic Membrane (Eosin Y)

The percentage of live spermatozoa was calculated using the Eosin test [49] to distinguish live from dead spermatozoa based on plasma membrane integrity. An amount of 5 µL of the sample was mixed with 5 µL of Eosin Y (0.5% wt/v) on a slide, then covered with a coverslip and read under a light microscope (Leica Microsystems), equipped with a camera at 40× magnification. At least 200 spermatozoa were counted in five different fields. Dead spermatozoa appeared stained or partially stained pink, while live spermatozoa were not stained.

### 2.5. DNA Fragmentation (SCD Test)

DNA fragmentation was evaluated using the protocol of Gosalvez et al. (2014) [33] with some modifications. The protocol is based on the Sperm Chromatin Dispersion (SCD) assay: a controlled process of nuclear protein removal followed by DNA denaturation. Therefore, normal spermatozoa have loops of DNA expanding from the head, resulting in scattered chromatin halos. An amount of 50 µL of the sample was mixed with 50 µL of 1% low-melting-point agarose (Biospa, Milan, Italy), previously melted at 100 °C for 5 min. An amount of 10 µL of the cell suspension was placed in the center of a slide, pre-coated with standard 0.65% agarose (Sigma Aldrich, Darmstadt, Germany) in PBS, and then covered with an 18 × 18 coverslip. The slide was transferred to a refrigerator at 4 °C for 5 min to solidify the agarose. Then, the coverslip was removed to put the slide for 2.5 min in lysis solution (2 M Nacl, 0.5% SDS, 0.01% TritonX, 0.2 M Tris-HCl, 0.02 EDTA, pH 7). The slide was washed with distilled water and left to incubate for 5 min. Next, the slide was treated with 70% ethanol for 2 min and 100% ethanol for another 2 min and allowed to air dry. Once dry, the slide was stained with Diff-Quick staining protocol, according to which1% Eosin Y (Bio Optica, Milan, Italy) was used for 2 min, followed by methylene blue (Bio Optica, Milan, Italy) for another 2 min. DNA halos were observed under an optical microscope (Nikon Eclipse E-200) in oil immersion at 100× magnification. At least 200 sperm were counted in five different fields.

### 2.6. Oxidative Stress

Oxidation of 2′,7′-dichlorofluorescin (DCFDA-H_2_) is widely used as a measure to detect the generation of reactive oxygen species (ROS). DFCDA-H_2_ is a probe bound to two acetyl groups that can cross membranes and, inside the cell, can be deacetylated by intracellular esterases that reduce it to Dichlorofluorescein (DCFH), a more hydrophilic and nonfluorescent compound. In the presence of ROS, DCFH is rapidly oxidized into the fluorescent compound DCF. Evaluation of reactive oxygen species production was performed following the protocol of Vignier et al. (2017) [50] with some modifications. Stock solution was prepared by dissolving the probe in dimethyl sulfoxide (DMSO, Sigma Aldrich) and stored in aliquots at −20 °C, thawed as needed. The probe was added to 150 µL of the sample to obtain a concentration of 10 µM. The sample was incubated at 18 °C for 30 min in the dark. Next, the sample was centrifuged at 2000 rpm for 15 min, and the pellet was resuspended in FSW. The sample was counterstained with 10 µL of 1 mM Hoechst 33342 (Thermo Fisher Scientific, Waltham, MA, USA) for 5 min and centrifuged at 2000 rpm for 15 min. The pellet was resuspended in 150 µL of FSW. Finally, the sample was smeared onto a slide and allowed to air dry. The slide was observed under a fluorescence microscope (Nikon Eclipse Ci) at 40× magnification. Spermatozoa with oxidative stress were DCF+/HOECHST+, while spermatozoa without stress were DCF-/HOECHST+. At least 200 spermatozoa were counted in five different fields. Images were analyzed using Nis Element software (version 5.20), through which fluorescence intensity was provided ed whose cut-off corresponds to 50 AU (Arbitrary Unit).

### 2.7. Fertilization Test

Following exposure, the spermatozoa were added to Petri dishes containing 15–20 eggs/mL (obtained by biopsy), the quality of which was verified via microscopic observation, in 15 mL of seawater to obtain a ratio of approximately 1:10 (eggs: sperm). To obtain this ratio, both gametes were previously counted using a Neubauer counting chamber. After 1 h, 2 mL from each plate were taken and placed in another Petri dish, where drops of 3.7% Formaldehyde (Bio-Optica) were added. The percentage of fertilized eggs was observed according to the presence of the fertilization membrane, the expulsion of the second polar globule, or the activated segmentation, via observation through an inverted microscope (Leica DIMIRB, Wetzlar, Germany) at 40× magnification. Three replicates were performed.

### 2.8. Statistical Analysis

Past 4.0 software was used to analyze data distribution (Shapiro–Wilk) and homogeneity of variances (Bartlett) and to highlight any statistically significant differences between the exposed groups and the control. Specifically, the one-way ANOVA test was performed, followed by Tukey’s test. The level of significance was set as α < 0.05, and the data were indicated with the symbol * if significant (*p* < 0.05) and with the symbol ** if highly significant (*p* < 0.01). All data are presented as mean ± standard deviation. Calculation of EC50, an index of effect size, was performed using AAT Bioquest. Lowest Observed Effective Concentration (LOEC) and the No Observed Effective Concentration (NOEC) were then deduced from the statistical analysis.

## 3. Results

### 3.1. Sperm Motility

**Figure 2:**
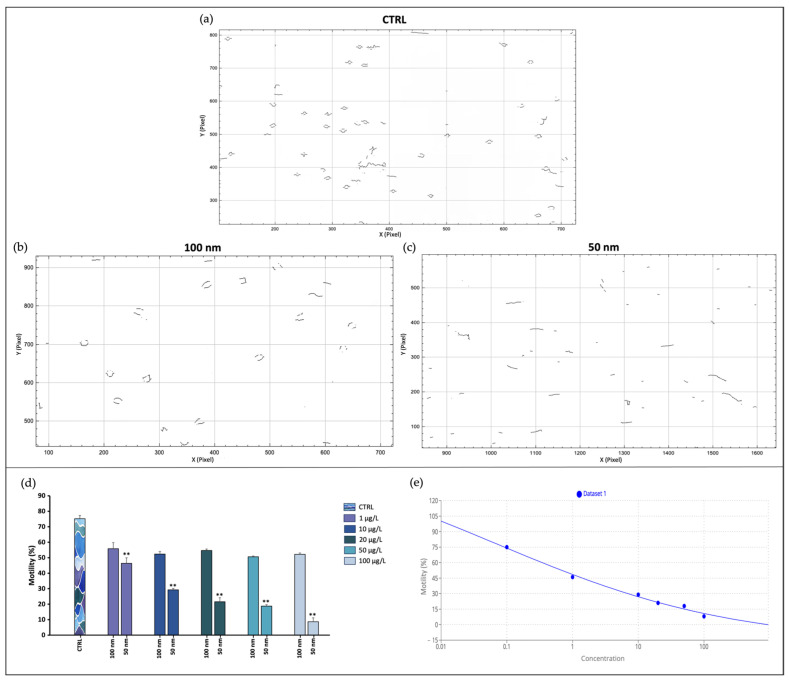
Movement analysis. (**a**) Pathway trajectory described by spermatozoa in the control group; (**b**) pathway trajectory described by spermatozoa exposed to 100 nm nPS-NH_2_; (**c**) pathway trajectory described by spermatozoa exposed to 50 nm nPS-NH_2_; (**d**) comparison analysis percentages of mobile spermatozoa after exposure to increasing concentrations of 50 and 100 nm nPS-NH_2_ compared with the control. Strong significant data are represented with the symbols ** (*p* < 0.01); (**e**) EC50 evaluation.

**Figure 3:**
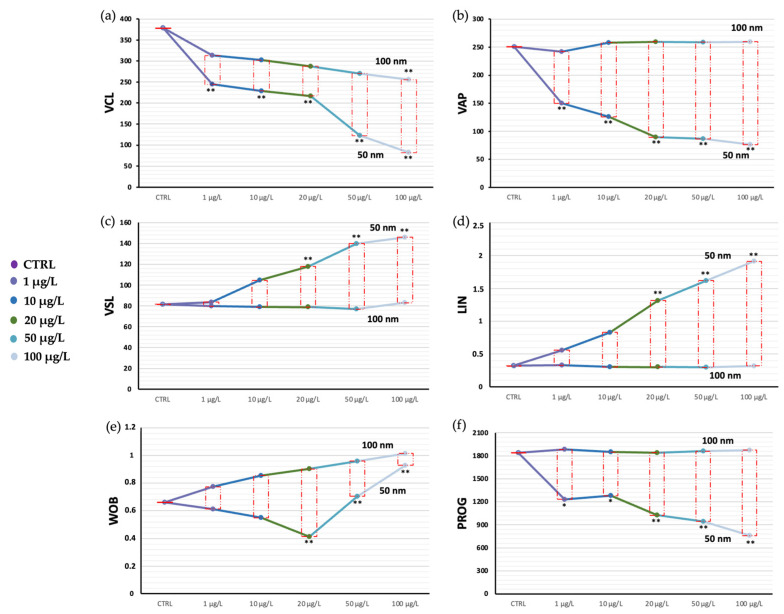
Comparison of descriptive values of the movement of spermatozoa exposed to increasing concentrations of 50 and 100 nm nPS-NH_2_ compared with control. (**a**) VCL; (**b**) VAP; (**c**) VSL; (**d**) LIN; (**e**) WOB; and (**f**) PROG. Significant data are represented with the symbols * (*p* < 0.05) and ** (*p* < 0.01).

Exposure of spermatozoa to increasing concentrations of nPS-NH_2_ with two different nanodiameters caused the reduction of the portion of motile spermatozoa in both cases, although a greater and statistically significant decline occurred in the case of the smaller nanoparticles. Additionally, from the analysis performed using ImageJ 1.44, descriptions of the spermatozoa’s spatial pathways, as well as predicted trajectory, velocity, and head oscillations were extracted (Figure 2). Again, decreases in VCL, VAP, and PROG emerged in samples exposed to the 50 nm nPS-NH_2_, correlated with the increase in tested concentration. VCL and VAP denoted a decrease in curvilinear velocity, while PROG denoted a decrease in spatial progression. These results were paralleled by increases in VSL, LIN, and WOB, especially for higher concentrations, whose values resulted in increased path linearity and head oscillation (Figure 3). In particular, up to the concentration of 20 µg/L, the oscillations tended to decrease, while at larger concentrations they tended to increase dramatically. The calculated EC50 corresponded to 0.07 µg/L. The LOEC was 1 µg/L, while the NOEC was not found. The values obtained are summarized in Table S1.

### 3.2. Integrity of Plasma Membrane

**Figure 4:**
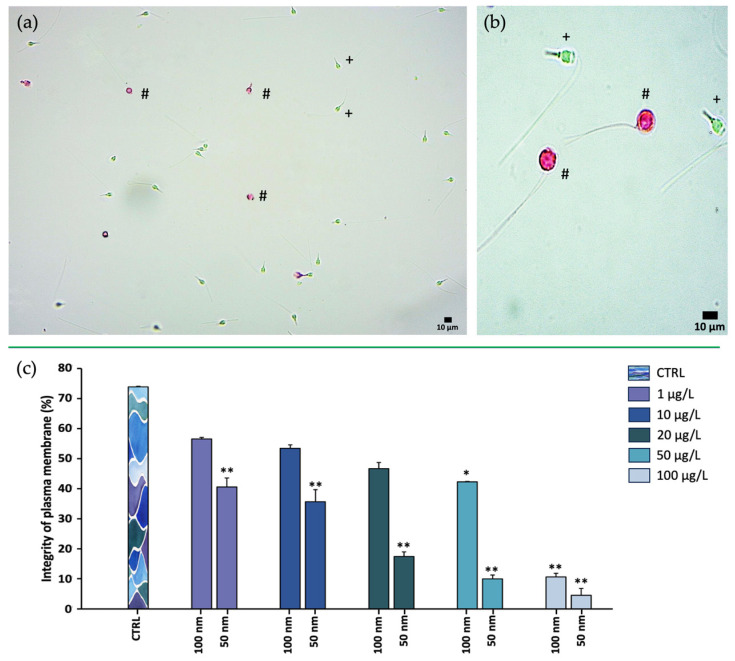
Eosin test. (**a**,**b**) Observation of spermatozoa, either intact (translucid, +) or damaged (pink, #), through optic microscope with 40× and 20× magnification, respectively; (**c**) comparison analysis percentages of intact spermatozoa exposed to increasing concentrations of 50 and 100 nm nPS-NH_2_ compared with the control. Significant data are represented with the symbols * (*p* < 0.05) and ** (*p* < 0.01).

**Figure 5:**
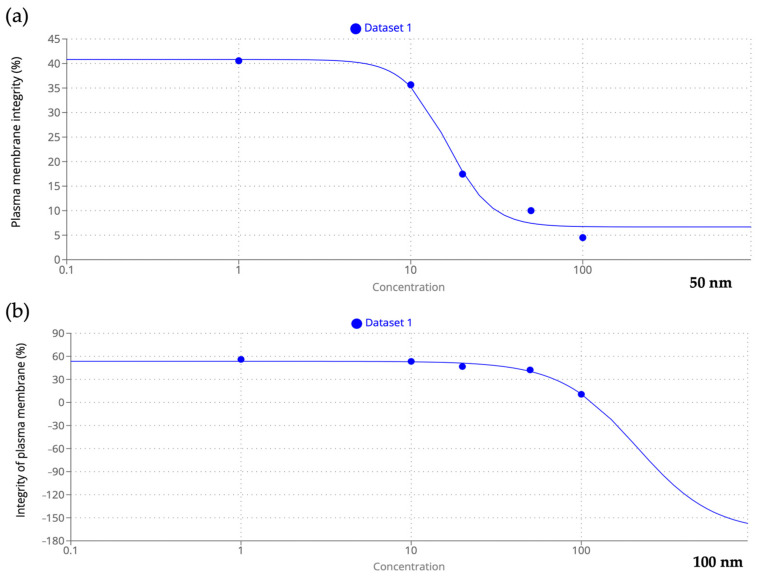
EC50 evaluation. (**a**) Relationship between membrane integrity and increased concentration of 50 nm nPS-NH_2_; (**b**) relationship between membrane integrity and increased concentration of 100 nm nPS-NH_2_.

**Figure 6:**
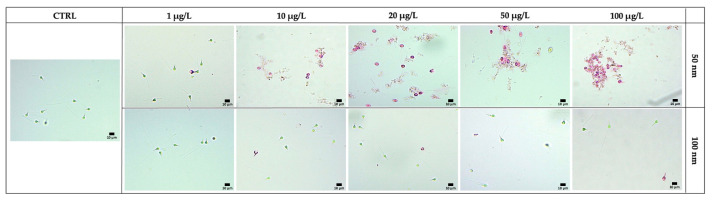
Agglomeration degree between spermatozoa exposed to increasing concentrations of 50 nm and 100 nm nPS-NH_2_ compared to the control group. Evident groups were noted from the concentration of 20 µg/L for samples exposed to 50 nm nPS-NH_2_.

The Eosin test allowed the distinction of viable transparent spermatozoa from nonviable spermatozoa in pink as shown in Figure 4. The results showed a negative correlation with increased concentration of both 50 and 100 nm nPS-NH_2_ compared to the control (73.88% ± 0.002); in the former category, even at the lowest concentrations, a significant decrease in membrane integrity was detected (40.56% ± 0.03 for 1 µg/L, 35.66% ± 0.04 for 10 µg/L, 17.45% ± 0.015 for 20 µg/L, 10% ± 0.012 for 50 µg/L, 4.50% ± 0.02 for 100 µg/L) while in the latter case, damage was evident only at the highest concentrations (42.30% ± 0.001 for 50 µg/L, 10.60% ± 0.012 for 100 µg/L). The greater toxicity of the smaller-diameter nPS-NH_2_ was also suggested by the results obtained from the EC50 calculation, according to which 50% of the adverse effects were obtained at a smaller concentration (16.21 µg/L) than for 100 nm nPS-NH_2_ (211.56 µg/L) (Figure 5). For 50 nm nPS-NH_2_, the LOEC corresponded to 1 µg/L, while the NOEC was not found. For 100 nm nPS-NH_2_, the LOEC and the NOEC were equal to 50 µg/L and 20 µg/L, respectively. Microscopic observation also revealed the presence of increasingly larger aggregates as the concentration increased in samples exposed to the smaller nPS-NH_2_. These results were not found in the samples exposed to the larger nPS-NH_2_ (Figure 6).

### 3.3. DNA Fragmentation

**Figure 7:**
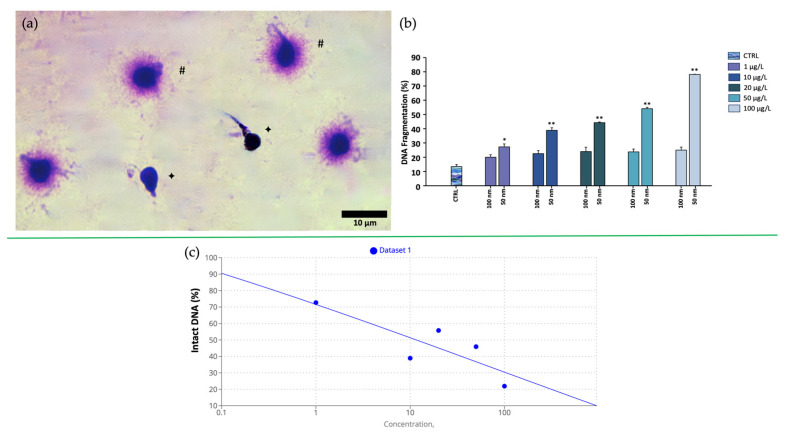
Halo test. (**a**) Observation of spermatozoa with intact DNA (with halo, #) and with damaged DNA (without halo, ◆) under optic microscope with 100× magnification (oil immersion); (**b**) comparison analysis of percentages of spermatozoa with fragmented DNA exposed to increasing concentrations of 50 and 100 nm nPS-NH_2_ compared with the control. Significant data are represented with the symbols * (*p* < 0.05) and ** (*p* < 0.01); (**c**) EC50 evaluation between decrease of intact DNA and increase of 50 nm nPS-NH_2_ concentration.

DNA fragmentation was assessed using the SCD test, which revealed spermatozoa with intact DNA via the presence of a halo around the head and gametes with fragmented DNA via the lack of the halo (Figure 7). Nonsignificant results were obtained for 100 nm nPS-NH_2_, while for 50 nm nPS-NH_2_ statistical analysis showed a reduction in the percentage of spermatozoa with intact DNA for all concentrations (13.44% ± 0.014 for CTRL, 27.30% ± 0.02 for 1 µg/L, 38.90% ± 0.018 for 10 µg/L, 44.24% ± 0.005 for 20 µg/L, 54.10% ± 0.01 for 50 µg/L, 78.10% ± 0.002 for 100 µg/L) whose data are highly significant. The calculated EC50 for 50 nm nPS-NH_2_ corresponds to 77.92 µg/L; the LOEC was equal to 1 µg/L, while the NOEC was not identified. 

### 3.4. Oxidative Stress

**Figure 8:**
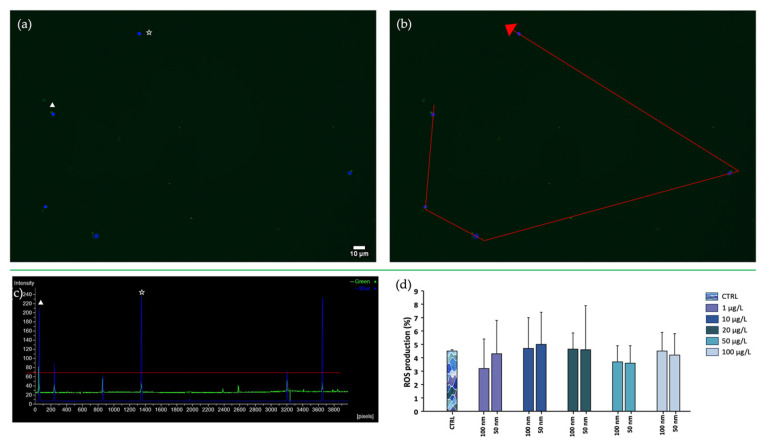
DCFH_2_-DA assay. (**a**) Observation of spermatozoa with oxidative stress (DCF+/HOECHST+, ▲) and without oxidative stress (DCF-/HOECHST+, ☆) under epifluorescent microscope with 40× magnification; (**b**) spermatozoa selection using Nis Element 5.20 software for image analysis; (**c**) fluorescence intensity plot with blue (DCF-/HOECHST+, ☆) and green (DCF+/HOECHST+, ▲) peaks; (**d**) comparison analysis of percentages of spermatozoa with oxidative stress exposed to increasing concentrations of 50 and 100 nm nPS-NH_2_ compared with the control. Nonsignificant data were found.

ROS production, assessed using the DCFH_2_-DA probe, allowed differentiation of spermatozoa with oxidative damage (green) from healthy spermatozoa (blue) (Figure 8). Nis Element software (version 5.20) analyzed the fluorescence emitted by gametes treated with the DCFH_2_-DA probe and contrasted with Hoechst 33342 dye, identifying the peaks emitted as shown in Figure 7. Nonsignificant results were reported regarding this parameter for both sizes of nPS-NH_2_; in fact, the percentages obtained in the exposed samples were comparable to those in the control.

### 3.5. Fertilization Toxicity Test

**Figure 9:**
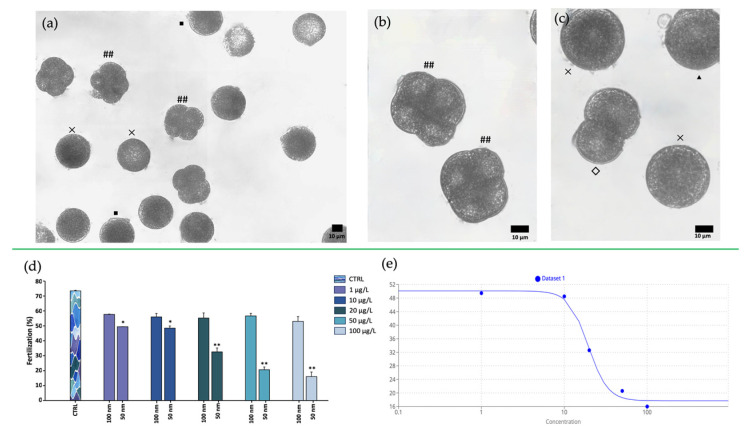
Fertilization assay. (**a**) Observation of samples under inverted light microscope with 20× magnification of unfertilized eggs (x), fertilized with fertilization membrane (■), and fertilized with active segmentation with 4 blastomeres (##); (**b**,**c**) observation of samples under inverted light microscope at 40× magnification of unfertilized eggs (x), fertilized with expulsion of the second polar globule(▲), and fertilized with active segmentation with 2 (◇) or 4 blastomeres (##); (**d**) fertilization rate of spermatozoa exposed to increasing concentrations of 50 and 100 nm nPS-NH_2_ compared with the control. Significant data are represented with the symbols * (*p* < 0.05) and ** (*p* < 0.01); (**e**) EC50 evaluation between fertilization and increase of 50 nm nPS-NH_2_ concentration.

The evidence of fertilized eggs (presence of fertilization membrane, expulsion of the second polar globule, two or four bastomere segmentation) was analyzed after 1 h of preparation of the mixture (Figure 9). The fertilizing capacity of the spermatozoa was negatively affected by the presence of the 50 nm nPS-NH_2_, which resulted in a significant reduction in the fertilization rate (49.45% ± 0.001 for 1 µg/L, 48.50% ± 0.014 for 10 µg/L, 32.60% ± 0.026 for 20 µg/L, 20.60% ± 0.019 for 50 µg/L, 16% ± 0.031 for 100 µg/L), compared with the control group (73.50% ± 0.003). In contrast, the exposure of the samples to the larger nPS-NH_2_ (100 nm) seemed to be irrelevant. The significant effect on fertilization was also underscored by the small EC50 value calculated, corresponding to 19.46 µg/L. The LOEC corresponded to 1 µg/L, while the NOEC was not found.

## 4. Discussion

The purpose of this experiment was to evaluate the effects of increasing concentrations of polystyrene nanospheres on the spermatozoa of *M. galloprovincialis*, a model aquatic organism. Indeed, the contamination of the seas and oceans by nanoplastics, pollutants of great concern, and their inevitable contact with organisms during their entire life cycle are now well known [51]. Moreover, exposure to such nanoparticles also involves gametes since in externally fertilized species, both oocytes and spermatozoa are released in seawater [52].

The sperm cell of bivalves is activated, following spawning, upon contact with seawater through the action of various chemical signals, including pH, ions, and cyclic nucleotides [53,54]. Once activated, the generation of an asymmetric oscillation of flagellum creates a curvilinear trajectory, interspersed with small linear segments [55,56]. The disturbance of the microenvironment, mainly caused by pollutants, could result in a change in swimming behavior [57]. In fact, in the samples exposed to 50 nm nPS-NH_2_, an alteration in the trajectory, which became less circular and more rectilinear, and in the oscillation of the head, which at the highest concentrations increased dramatically, were noted. Under natural conditions, bivalve spermatozoa orient their trajectory on a straight path upon hyperactivation. This process is generated by chemoattractants released from the egg cell, which, after being recognized by specific receptors, trigger the opening of calcium channels, leading to increased speed and oriented movement [58,59,60,61]. Chemotaxis involves the release by the egg cell of chemical signals that direct the path of the spermatozoa, determining a substantial switch in their movement. As previously observed, with the lack of chemotactics the spermatozoa described a circular path, whereas in the presence of the egg cells they acquired a distinct straight direction [40,62]. To date, although chemotaxis in *M. galloprovincialis* has been documented, the identification of chemoattractants remains a field of research to be further explored. The results obtained would suggest that smaller nanoplastics may share some degree of chemical and structural similarity with such molecules. Settling at the level of the acrosome suggests the affinity of nanoplastics for receptors in the acrosomal membrane, as demonstrated by González-Fernández et al., 2018, [40] which prompts our interest in identifying these molecules and evaluating the similarity with nanoplastics to study the possible interaction with specific sperm receptors, indispensable for hyperactivation.

In this experiment, a decrease in velocity also occurred. This evidence could be explained by the steric obstruction due to 50 nm nPS-NH_2_, which aggregate, forming larger complexes that force the spermatozoa into a more difficult and energy-consuming movement. Despite the adaptive strategies of the spermatozoon, including increasing head oscillations to overcome possible obstacles, the physical presence of aggregates could limit sperm movement and consequently the success of fertilization [63]. As observed in the present study, motility decreased in relation to the increasing concentration, parallel to aggregate formation. In line with our observations, about the reduction in motility of exposed spermatozoa, a study reported that testing 50 nm amino-modified polystyrene particles with a concentration of 10 μg/mL, high spermiotoxicity, characterized by a decrease in the percentage of motile sperm (−79%) and velocity (−62%) compared to control spermatozoa, was observed. The reduction in motility was partly explained by the formation of homo- or heteroaggregates of plastic nanoparticles. In fact, this study showed, through both confocal microscopy and SEM, the existence of a myriad of spermatozoa trapped within large aggregates of plastic particles [64]. Interestingly, the concentrations in the present study were significantly lower, but nonetheless the same toxic effects were found. Canesi et al., 2015, and Della Torre et al., 2014, also reported the tendency of nanoplastics to agglomerate in seawater [65,66]. The phenomenon depended greatly on the charges, the functional groups, and the sizes of the molecules [67]. In particular, smaller and positively charged compounds (such as in the amine group used in the present study) might aggregate faster than larger molecules or with a negative charge (e.g., carboxylic group). This possibly explains why agglomeration was only reported for the 50 nm nanoplastics in the present study. In this context, the aggregation kinetics of polystyrene nanoplastics requires further investigation. 

It is important to emphasize how the surrounding environment, disrupted by the presence of pollutants, can affect the kinematic characteristics of swimming [40]. Organic pollutants, in fact, could alter the pH, salinity, viscosity, and osmolarity of the water and the concentration of ions, creating a micro-ecosystemic equilibrium that complicates the exchange of information between the spermatozoon and the external environment. The parameter most affected would be precisely the activation and kinematics of movement [68,69]. The change in the chemical and physical characteristics of water in relation to the presence of increasing concentrations of pollutants certainly requires attention and the setup of trials that clarify the role of nPS-NH_2_. 

Physical contact between cells and nanoparticles could affect the vitality rate due to mechanical damage to the plasma membrane leading to cell necrosis. The Eosin test, in fact, identified nonviable spermatozoa with lesions and ruptures of the plasma membrane. Several studies in the literature noted the interaction between nPS-NH_2_ and bivalve spermatozoa. In the study of Tallec et al. (2020) [39], oyster spermatozoa were exposed for 1 h to various doses (from 0.1 to 25 μg/mL) of amino- or carboxy-modified 50 nm nPS-NH_2_. Microscopy detected the positioning of the nPS-NH_2_, confirming adhesion of the particles to sperm membranes, but no translocation within the cells. This lack of internalization could be explained by some properties of germ cells; in fact, spermatozoa do not exhibit endocytosis processes important for the internalization of nanoparticles [70]. Despite the very low level of internalization by sperm cells, cationic nanoparticles interact more readily with negatively charged membrane residues, triggering internalization to balance the charge, but at the same time triggering instabilities and subsequent membrane ruptures [71,72].

The possibility of nPS-NH_2_ entry into the cell, through ruptures, and the possible establishment of membrane interactions could explain the increased DNA damage observed in samples exposed to 50 nm nPS-NH_2_. The genotoxicity of nPS-NH_2_ on the spermatozoa of bivalves has not yet been adequately investigated. Data in the literature focus especially on echinoderm spermatozoa and suggest the sensitivity of the genome of aquatic spermatozoa to the presence of the plastics [73]. 

Another endpoint analyzed in our study was the overexpression of ROS, an index of the oxidative stress state of cells. It is known that an imbalance between antioxidant and oxidant systems causes the uncontrolled release of radical molecules, which can react with macromolecules by oxidizing and degrading them through lipid peroxidation phenomena [74,75]. In our study, no evidence of increased ROS after 30 min of exposure was found, although in another study, significant ROS generation and subsequent oxidative stress were described after 5 h of exposure [55]. In this case, time may be the main variable to consider. 

It is known how the worsening of the above parameters is involved in the failure of fertilization [74]. From the evidence obtained, it was evident that the 50 nm nanoplastics resulted in the reduction (by 33% for 1 µg/L and for 10 µg/L, 56% for 20 µg/L, 72% for 50 µg/L, 78% for 100 µg/L) in the percentage of fertilized eggs compared to the control, proportional to the increase in tested concentration. One of the parameters most closely related to fertilization was motility, as well as membrane integrity. From emerging data in the literature, DNA fragmentation, on the other hand, does not appear to lead to fertilization failure, but rather to the development of abnormal embryos and larvae with a very low chance of survival [76,77,78].

The size-related toxicity of nPS-NH_2_ was also confirmed by calculating the EC50 value. Lower values were obtained for smaller nanoplastics. In addition, this calculation also helped to identify which parameters are more susceptible, as adverse effects were achieved at lower concentrations. The most affected parameter was motility for which the effect of nanoplastics on 50% of the sample was already apparent at the concentration of 0.07 µg/L, followed by membrane integrity (16.21 µg/L), fertilization success (19.46 µg/L), and DNA fragmentation (77.92 µg/L). Finally, deduction of LOEC and NOEC values contributed to clarifying the concentrations responsible for the onset of damage. In the case of 50 nm nanoplastics, the lowest concentration tested (1 µg/L) resulted in high damage for all parameters tested, except for ROS, for which further studies are needed to establish a threshold below which these substances do not exert any harmful action. For the 100 nm nanoplastics, the LOEC and the NOEC, for the viability parameter, were equal to 50 µg/L and 20 µg/L, respectively. It might be desirable, therefore, to investigate this range to determine a possible threshold value more accurately.

## 5. Conclusions

In conclusion, nanoplastics pollution in the seas and oceans is correlated to reduction of organism fertility. nPS-NH_2_ can not only physically obstruct the union of sperm and egg cell, but also chemically interact with spermatozoa membranes, causing structural damage to organelles and altering metabolic functions. To fully understand the role of nanoplastics in altering the marine microenvironment, it is imperative to deepen our insights not only into the interaction with gametes, but also into their influence on the chemical and physical parameters of water, such as pH, concentration of oxygen, and carbon dioxide, as well as ionic concentration. Future prospects must be directed toward understanding all parameters (biotic and abiotic) intimately related to the balance of biological systems, adults, embryos, and gametes in order to adopt the most appropriate strategies to reduce the negative impact of nanoplastics in the marine environment.

## Figures and Tables

**Table 1:** Summary of studies that evaluated the effects of polystyrene nanoplastics on bivalve spermatozoa.

Model Organism	Diameter of Nanoplastics	Concentration	Tested Parameters	Results	Author
*Cassostrea gigas*	50 nm nPS-NH_2_ 50 nm nPS-COOH	From 0.1 µg/mL to 0.25 µg/mL	Motility Velocity Fertilization	Higher toxicity of nPS-NH_2_ with reduction of analyzed parameters	Tallec et al., 2020 [39]
*Cassostrea gigas*	100 nm nPS-NH_2_ 100 nm nPS-COOH	0.1, 1, 10 and 100 mg/ L	Motility Vitality Oxidative stress	Increased oxidative stress at the higher concentrations of nPS-COOH	González-Fernández et al., 2018 [40]
*Cassostrea gigas*	50 nm nPS-NH_2_ 50 nm nPS-COOH	0.1, 1, 10 and 25 μg/mL	Fertilization Embryogenesis Metamorphosis	Higher toxicity of nPS-NH_2_ with reduction of fertilization rate	Tallec et al., 2018 [41]
*Mytilus galloprovincialis*	Environmental micro- and nanoplastics	1, 10, 50, and 100 μg/L	Motility Vitality Oxidative stress Mitochondria DNA integrity Apoptosis	Decrease in all parameters tested	Romdhani et al., 2023 [42]
*Tegillarca granosa*	500 nm and 5 μm nPS-NH_2_	0.26 and 0.69 mg/L	Motility Viability DNA integrity Apoptosis Fertilization	Increase in DNA fragmentation and decline in fertilization rate	Shi et al., 2022 [43]

## Data Availability

Original data are available upon request.

## Supplementary Materials

The following supporting information can be downloaded at: https://www.mdpi.com/article/10.3390/toxics11110924/s1, Table S1: Properties and percentages of mobile spermatozoa after exposure to 50 nm and 100 nm of NPs at increasing concentration.
