# Supplementary material for: Polystyrene Nanoplastics in Aquatic Microenvironments Affect Sperm Metabolism and Fertilization of Mytilus galloprovincialis (Lamark, 1819)
Source: Toxics. 2023 Nov 11;11(11):924. doi: 10.3390/toxics11110924 (PMC10675086; doi:10.3390/toxics11110924)
Supplement: Supplementary file 1 [file toxics-11-00924-s001.zip › toxics-2697258-supplementary.pdf]

Polystyrene Nanoplastics in Aquatic Microenvironments Affect Sperm Metabolism and Fertilization of *Mytilus galloprovincialis* (Lamark, 1819)

Martina Contino [1,*], Greta Ferruggia [1], Stefania Indelicato [1], Roberta Pecoraro [1], Elena Maria Scalisi [1], Antonio Salvaggio [2] and Maria Violetta Brundo [1]

1. Department of Biological, Geological and Environmental Sciences, University of Catania, Via Androne 81, 95124 Catania, Italy; greta.ferruggia@phd.unict.it (G.F.); stefania.indelicato@phd.unict.it (S.I.); roberta.pecoraro@unict.it (R.P.); elenamaria.scalisi@unict.it (E.M.S.); mariavioletta.brundo@unict.it (M.V.B.)
2. Zooprophylactic Institute of Sicily "A. Mirri", Via Gino Marinuzzi, 3, 90129 Palermo, Italy; antonio.salvaggio@izssicilia.it

* Correspondence: martinacontino@phd.unict.it; Tel.: +39-3271681121

Supplementary materials

Table S1. Properties and percentages of mobile spermatozoa after exposure to 50 nm and 100 nm of NPs at increasing concentration.

		CTRL	1 µg/L	10 µg/L	20 µg/L	50 µg/L	100 µg/L
Motility (%)	50 nm	75.23%±0.02	46.42%±0.03	29.30%±0.01	21.59%±0.02	18.76%±0.01	8.66%±0.02
	100 nm		55.86%±0.04	52.36%±0.02	54.65%±0.01	50.63%±0.004	52.18%±0.008
VCL	50 nm	378.76±0.015	244.53±0.01**	228.96±0.03**	216.93±0.032**	122.74±0.022**	82.23±0.02**
	100 nm		313.15±0.002	302.12±0.025	287.57±0.012	270.14±0.024	255.6±0.01
VAP	50 nm	250.84±0.032	149.78±0.023**	125.84±0.001**	89.61±0.014**	86.47±0.034**	76.36±0.03**
	100 nm		242.29±0.14	258.22±0.22	259.45±0.01	259.06±0.09	259.45±0.04
VSL	50 nm	81.56±0.023	83.70±0.052	104.6±0.034	117.77±0.25**	139.82±0.15**	146.03±0.12**
	100 nm		79.72±0.075	78.93±0.03	78.91±0.01	76.88±0.36	83.13±0.56
LIN	50 nm	0.32±0.034	0.56±0.32	0.83±0.56	1.31±0.15**	1.62±0.1**	1.91±0.27**
	100 nm		0.33±0.36	0.3±0.027	0.3±0.15	0.29±0.12	0.32±0.08
WOB	50 nm	0.66±0.025	0.612±0.01	0.55±0.037	0.41±0.28**	0.7±0.09**	0.93±0.07**
	100 nm		0.77±0.02	0.85±0.08	0.9±0.06	0.96±0.56	1.01±0.67
PROG	50 nm	1840.29±0.15	1232.92±0.03*	1279.31±0.09*	1025.37±0.15**	944.38±0.18**	759.44±0.11**
	100 nm		1882.155±0.04	1848.3±0.044	1739.32±0.033	1861.13±0.022	1905.06±0.22

All data are presented ad mean ± standard deviation. Significant data are represented with the symbol * ($p < 0.05$) and ** ($p < 0.01$) and are highlighted in yellow.
